# Access to Drugs for Treatment of Noncommunicable Diseases

**DOI:** 10.1371/journal.pmed.1001485

**Published:** 2013-07-23

**Authors:** Thomas J. Bollyky

**Affiliations:** Council on Foreign Relations, Washington, D.C., United States of America

## Abstract

Thomas Bollyky explains the emerging controversy over patented medicines for noncommunicable diseases and their affordability in developing countries. Another transformation in global health is required that focuses on low-cost interventions and patient-centered, rather than country-focused, strategies.

*Please see later in the article for the Editors' Summary*

**Summary Points**

- A decade ago, the HIV/AIDS treatment-access crisis helped elevate infectious diseases as a foreign policy issue and mobilized billions in global health aid.
- A new controversy over patented medicines and their affordability in developing countries is emerging, this time over noncommunicable diseases (NCDs).
- Conflicts over patented NCD medications are likely to increase, with potential adverse consequences for patients, drug firms, and developed and developing country governments alike.
- The intergovernmental institutions designated to address trade and global health concerns are unlikely to resolve these conflicts and alternatives to intellectual property have not attracted significant donor and multilateral support.
- Addressing the NCD treatment-access crisis will require another transformation in global health, this time focusing on low-cost interventions and patient-centered, rather than country-focused, strategies.

A decade ago, bitter disputes over access to patented HIV/AIDS medicines in developing countries transformed global health, elevating infectious diseases as a foreign policy concern and helping to mobilize billions of dollars to research and distribute new therapies to meet the needs of the world's poor. Now, a new fight over treatment access looms. India, China, and other middle-income countries have taken measures to circumvent patents on medicines for diabetes, cancer, and cardiovascular and chronic respiratory illnesses—the noncommunicable diseases (NCDs) increasing most rapidly in low- and middle-income countries. Addressing this latest treatment-access crisis will require another transformation in global health, this time focusing on NCDs, low-cost interventions, and patient-centered strategies.

## The Crisis over Access to HIV/AIDS Treatment

International disputes over treatment access in developing countries are a relatively new phenomenon. Historically, few effective therapies have existed for use in these countries. There was little investment in new treatments for the infectious and neglected diseases that disproportionately affect developing countries because most people who suffer from them are desperately poor. Many of the existing therapies were developed for veterinary use or dated back to the colonial era [1].

Two developments sparked the international controversy over access to medicines in the late 1990s. First, global trade talks established the World Trade Organization (WTO) and its Agreement on Trade-Related Aspects of Intellectual Property Rights (TRIPS) in 1995, which mandated minimum standards of intellectual property (IP) protection, including pharmaceutical patents, in member countries. Second, new, life-saving antiretroviral medicines (ARVs) emerged for HIV/AIDS, a disease that gained prominence first in Europe and the United States but has since disproportionately plagued developing countries, particularly in sub-Saharan Africa.

Pharmaceutical companies, concerned about undercutting sales in developed country markets, adopted internationally consistent prices for their ARVs. In 1998, ARVs cost more in South Africa, on a per capita GDP-adjusted basis, than in Sweden or the United States [2]. Just 10,000 out of the almost four million South Africans living with HIV/AIDS had access to the treatment that could save their lives [3]. In Brazil and South Africa, patients and advocates protested an international IP system that prioritized profits over patients and failed to incentivize research to meet their health needs. Pharmaceutical companies and their developed country supporters defended the need for strong IP protection to sustain future innovation.

Protests spread, disrupting international HIV/AIDS meetings and the 1999 Seattle WTO Ministerial Conference [4]. Trade disputes and court battles erupted over compulsory licenses, a tool provided in the TRIPS agreement for governments to license a patent without the consent of its owner [5]. Between 2001 and 2005, WTO members issued 17 compulsory licenses on pharmaceutical patents, the vast majority involving ARVs [6]. Popular support for the pharmaceutical industry and international trade plummeted.

Amid the HIV/AIDS treatment-access crisis, investment in addressing the health needs of developing countries increased dramatically. International development assistance for health grew more than 10% annually from 2001 to 2010, from US$10.8 billion to US$28.2 billion [7]. Annual funding for R&D on HIV/AIDS, malaria, TB, and other infectious diseases rose 30-fold, to more than US$3 billion [8]. The US government, the Bill & Melinda Gates Foundation, and other donors established the Global Alliance for Vaccines and Immunization (GAVI) in 2000 [9]; the Global Fund to Fight AIDS, TB, and Malaria (Global Fund) in 2002 [10]; and the US President's Emergency Plan for AIDS Relief (PEPFAR) in 2003 [11]. These programs now deliver drugs and vaccines to millions in developing countries. Pharmaceutical firms and universities donated or voluntarily licensed their IP relevant to diseases and products for which there is little demand in affluent markets [12]. Competition and voluntary price cuts reduced the cost of ARVs in poor countries from US$12,000 per year in 2001 to US$200 per year in 2005 [13]. With expanded access to treatment and cheaper ARVs, the number of compulsory licenses on patented medicines declined dramatically between 2006 and 2011 [6].

A variety of motivations fueled this surge in global health investment, including humanitarian concerns and the launch of the Millennium Development Goals (MDGs) in 2000. The catalyst, however, was the emergence of the HIV/AIDS treatment-access crisis. Infectious and neglected diseases had long plagued developing countries without generating a significant international humanitarian response. The MDGs on other issues attracted far fewer resources than the goals on HIV/AIDS, malaria, and maternal health. GAVI and PEPFAR were launched prior to the MDGs or pointedly without reference to them [14]. The HIV/AIDS treatment-access crisis brought international attention to a long-standing problem—international systems for IP, medical R&D, and trade were not responding to the health needs of developing countries—and helped to motivate donors and the private sector to do more to resolve it.

## The New Treatment-Access Crisis over NCDs

In 2012, a new controversy over patented medicines and their affordability in developing countries emerged, this time over NCDs. Earlier this year, India's highest court rattled the multinational drug industry by refusing to grant a patent on a modified version of Gleevec, an anti-leukemia drug [15]. India also issued a compulsory license on a treatment for liver and kidney cancer and announced its intention to issue licenses on two breast cancer drugs and a leukemia treatment [16]. Indonesia issued compulsory licenses on seven drugs, including a treatment for liver cancer–causing hepatitis B [17]. China and the Philippines amended their patent laws, making it easier to issue compulsory licenses for medicines [18],[19]. Four trends are driving these moves and explain their increasing frequency in middle-income countries.

First, the toll of NCDs is increasing dramatically in developing countries, particularly in middle-income countries, but global health investment has not kept pace. According to the World Health Organization (WHO), 80% of deaths from NCDs now occur in low- and middle-income countries, up from 40% percent in 1990 [20]. By 2030, NCDs will be the leading cause of death and disability in every region of the world [20]. A recent report by Harvard University and the World Economic Forum projects that over the next two decades, NCDs will inflict US$14 trillion in economic losses on the developing world [21]. Yet only US$185 million of the US$28.2 billion spent globally on development assistance for health in 2010 was dedicated to NCDs [7].

Second, access to effective treatment for NCDs, patented or otherwise, remains limited in many low- and middle-income countries [22]. Most drug purchases still occur out-of-pocket and are beyond the means of many poor households [23]. NCDs that are preventable, such as cervical cancer, or treatable, such as juvenile diabetes, are often death sentences in developing countries [20].

Third, consensus on a sustainable model for pricing pharmaceuticals for middle-income country markets remains elusive. On one hand, these are emerging economies with resources that many drug firms argue should be paying their fair share for pharmaceutical innovation. China and India have space programs and international aid agencies. On the other hand, 70% of the world's population that survives on less than US$2 per day lives in middle-income countries such as China, India, and Nigeria [24]. Extending health care to these populations is an enormous undertaking that will require time and tremendous resources.

Fourth, middle-income countries have both health and industrial policy reasons for encouraging domestic production of NCD therapies. Mexico, China, and India are expanding public health spending on medicines, but costs are rising fast. IMS Health projects pharmaceutical spending in emerging economies to more than double by 2016, to US$300 billion annually [25]. Compulsory licensing and stricter patentability standards allow domestic manufacturers to produce lower-cost versions of patented NCD medications and break into lucrative therapeutic areas, such as oncology, in which multinational drug firms are heavily invested [25].

As the use of measures to circumvent patents on NCD drugs increases so will the opposition to them. The multinational pharmaceutical industry has staked its future on these diseases and emerging markets. United States, Europe, and other developed countries are likewise heavily invested in the international IP protection system. The flexible IP policies applied to HIV/AIDS and other infectious diseases that disproportionately affect poor countries are unlikely to be easily extended to large, emerging economies and the NCDs that plague both rich and poor patients alike.

In this new fight over treatment access, there may be many losers. Multinational firms may forego developing or registering lifesaving NCD therapies for use in countries with a high risk of compulsory licensing. The US Congress is reportedly considering excluding India from its Generalized System of Preferences (GSP) program, through which India exported US$4.5 billion goods duty-free to the United States in 2012 [26]. If patients are pitted against patents, international support for IP protection—upon which drug firms and many other developed country industries now heavily rely—will again diminish.

The WHO, WTO, and the other intergovernmental institutions designated to address trade and global health concerns are unlikely to resolve the bitter disputes emerging over access to NCD medicines. Negotiations to amend the TRIPS Agreement in 2003 to make compulsory licensing more usable by low-income countries were deeply contentious and produced a solution so administratively complex that it has been used only once [27]. Talks at the WHO on IP alternatives like prize funds and R&D treaties have likewise gone nowhere. Unless other strategies can meet developing country needs on NCDs, this latest fight over access to medicines is likely to escalate, particularly in middle-income countries.

## Patients Versus Patents Should Be the Precursor to Action on NCDs

The international systems supporting trade, IP, and medical R&D have spurred pharmaceutical innovation and economic growth, but can function only with the support of their constituents. In the HIV/AIDS crisis, the actors that have benefited most from those systems—developed country governments and industry—found IP-consistent ways to meaningfully address the legitimate concerns of developing country governments and patients. The emerging fight over patented NCD therapies in developing countries will only be resolved with similar actions. Fortunately, addressing NCD treatment demands in developing countries should not require the massive resources mobilized for HIV/AIDS. The following low-cost strategies provide a way forward.

### 

#### Frugal innovation

There is tremendous investment in NCD R&D generally, but little targets drugs and diagnostics that would be usable in low-resource settings with limited health infrastructure. Pharmaceutical firms, universities, and developed country governments should adopt the same humanitarian IP licensing policies for these purposes as they have applied to neglected diseases [28]. Modest donor support is required for product-development partnerships, such as the international organization PATH, which is working to adapt existing treatments and diagnostics for use in low-infrastructure settings. Expanding treatment platforms such as GAVI and the Global Fund would enable delivery of these frugal innovations.

#### Financial incentives and pooled procurement

Many effective therapies for NCDs— ACE inhibitors, beta blockers, and insulin—are off-patent, but unavailable in many developing countries. These products are among WHO's best-buy strategies for addressing NCDs, but many lack international suppliers or are difficult for still-nascent developing country regulatory authorities to oversee [29]. Financial incentives, such as advance market commitments, and pooled procurement may be required to scale up manufacturing of these treatments, ensure their affordability, and facilitate their delivery to developing country patients. Sophisticated national regulatory authorities should help ensure the quality and safety of these, as is currently done with PEPFAR.

#### Intra-country differential pricing

Pharmaceutical pricing for developing countries would be more sustainable if predicated on the income status of the patient, rather than the country involved. Pharmaceutical companies should participate in access programs that charge different prices for drugs that will be sold to higher-income patients, covered by corporate insurance plans or treated in private hospitals, than to lower-income patients treated in public clinics or resource-poor, rural settings. For some treatments, it may be necessary to grant licenses to emerging country generic manufacturers better able to meet the low-cost, high-volume needs of the poor patients that multinational firms are ill-suited to serve. Pharmaceutical firms should adopt differentiated packaging to help prevent the arbitrage of products. Participating developing countries should commit contractually to ensuring that the product is only used in the market segment for which it is intended [30].

## Conclusion

A decade ago, a crisis over access to HIV/AIDS treatment transformed the global response to that disease and the other infectious and neglected diseases disproportionately affecting the world's poor. Expanding access to treatment for those diseases saved the lives of millions and stabilized support for the international trade and IP systems that have since benefited developed and many emerging economies alike. Now, a new treatment-access crisis rages over NCDs. The global community must again respond to the legitimate treatment needs of developing countries and their patients grappling with this latest epidemic. The practical, scalable strategies outlined here would provide the means for doing so even in these austere times.
